# Polar body based aneuploidy screening is poorly predictive of embryo ploidy and reproductive potential

**DOI:** 10.1007/s10815-014-0293-1

**Published:** 2014-08-09

**Authors:** C. N. Salvaggio, E. J. Forman, H. M. Garnsey, N. R. Treff, R. T. Scott

**Affiliations:** 1 Division of Reproductive Endocrinology, Department of Obstetrics, Gynecology and Reproductive Sciences, Rutgers-Robert Wood Johnson Medical School, New Brunswick, NJ 08901 USA; 2 Reproductive Medicine Associates of New Jersey, 140 Allen Road, Basking Ridge, NJ 07920 USA

**Keywords:** Preimplantation genetic screening (PGS), Polar bodies, Aneuploidy, Blastocyst, SNP array

## Abstract

**Purpose:**

Polar body (polar body) biopsy represents one possible solution to performing comprehensive chromosome screening (CCS). This study adds to what is known about the predictive value of polar body based testing for the genetic status of the resulting embryo, but more importantly, provides the first evaluation of the predictive value for actual clinical outcomes after embryo transfer.

**Methods:**

SNP array was performed on first polar body, second polar body, and either a blastomere or trophectoderm biopsy, or the entire arrested embryo. Concordance of the polar body-based prediction with the observed diagnoses in the embryos was assessed. In addition, the predictive value of the polar body -based diagnosis for the specific clinical outcome of transferred embryos was evaluated through the use of DNA fingerprinting to track individual embryos.

**Results:**

There were 459 embryos analyzed from 96 patients with a mean maternal age of 35.3. The polar body-based predictive value for the embryo based diagnosis was 70.3 %. The blastocyst implantation predictive value of a euploid trophectoderm was higher than from euploid polar bodies (51 % versus 40 %). The cleavage stage embryo implantation predictive value of a euploid blastomere was also higher than from euploid polar bodies (31 % versus 22 %).

**Conclusion:**

Polar body based aneuploidy screening results were less predictive of actual clinical outcomes than direct embryo assessment and may not be adequate to improve sustained implantation rates. In nearly one-third of cases the polar body based analysis failed to predict the ploidy of the embryo. This imprecision may hinder efforts for polar body based CCS to improve IVF clinical outcomes.

## Introduction

Improving IVF outcomes is a critical focus of contemporary reproductive medicine. Given the direct, causal relationship between chromosomal aneuploidy and human pregnancy loss, congenital birth defects, and IVF failure, there is great interest in the clinical application of aneuploidy screening to improve IVF outcomes. A validated single nucleotide polymorphism (SNP) array-based technology was previously developed to comprehensively and accurately assess the chromosomal status of embryos [1]. This technology has proven to be highly predictive of the reproductive potential of human embryos [2] and has demonstrated clinical efficacy in a randomized controlled trial [3]. Alternate methods of comprehensive chromosome screening (CCS), including quantitative real-time PCR, have also been developed and have yielded excellent outcomes in a retrospective study [4] and in randomized controlled trials [5, 6].

While there have been significant advances in screening technologies, the question remains as to what stage in embryonic development, from the oocyte to the expanded blastocyst, is the most appropriate for a biopsy to obtain DNA for aneuploidy screening. Although all randomized controlled trials of CCS reported to date have involved testing at the blastocyst stage of development [3, 5–7], polar body based aneuploidy screening remains an option under consideration [8]. Through examination of both polar bodies, it is possible to determine whether the chromosomes segregated correctly during meiosis. Furthermore, polar body biopsy has been advocated as a less invasive procedure than embryo biopsy [9], since polar bodies are naturally extruded during development.

Given that maternal meiotic error is the major contributor to embryonic aneuploidy [10], the selective transfer of embryos derived from oocytes that correctly segregated chromosomes during meiosis has been hypothesized to improve clinical outcomes [11]. In fact, the European Society of Human Reproduction and Embryology (ESHRE) PGS Task Force has launched a multicenter randomized controlled trial to characterize the utility of polar body aCGH [12, 13]. However, owing to the indirect nature of polar body based aneuploidy screening, there is concern regarding its ability to accurately predict the chromosomal status of the embryo [14–16] and its reproductive potential. We sought to address these concerns by comparing sequential biopsies of polar bodies and ensuing embryos to determine the predictive value of polar body testing for embryo ploidy and to perform a prospective blinded nonselection analysis of the predictive value of polar body based screening for delivery after embryo transfer.

## Materials and methods

### Population and ART cycles

The study population consisted of 96 couples attempting assisted conception, in which the female partner was aged 24 to 42 years. The mean maternal age was 35.3 ± 4.6 years and the mean paternal age was 38.8 ± 5.8 years. Cycles using an oocyte donor were included and the age of the donor was used. All patients were required to have a basal antral follicle count of ≥8 and a serum day 3 FSH concentration of <12 IU/L. Couples with 2 or more failed IVF cycles, i.e. no delivery from the entire cohort of fresh and frozen embryos, were excluded. Couples with a history of endometrial insufficiency, chronic anovulation secondary to polycystic ovarian syndrome or with severe male factor infertility requiring surgical sperm extraction were also excluded. Both partners were required to have peripheral blood samples collected, which allowed for isolation of parental DNA. The acquisition of parental DNA was necessary for accurate DNA fingerprinting of the embryos and infants as previously described [17].

All patients underwent routine IVF stimulation, as determined by the patient’s primary physician. There were no restrictions on the type of follicular stimulation used in the study. All aspects of retrieval and oocyte recovery were performed using established routine laboratory procedures and have been previously reported [2].

### Experimental design

The data in this investigation was extracted from a nonselection study, which aimed to validate the accuracy of DNA fingerprinting using polar bodies and embryonic cells. The study was registered with ClincalTrials.gov under the identifier NCT01219517. The specific study design has been previously reported [2]. In short, all embryos in the study underwent the same triple-biopsy procedure. Metaphase II oocytes underwent first polar body biopsy following oocyte retrieval and intracytoplasmic sperm injection (ICSI). At the time of fertilization check the following morning, normally fertilized oocytes with 2 pronuclei underwent a second polar body biopsy. Finally, embryos were biopsied prior to transfer on day 3 or day 5, as previously described [2]. Genetic results were not available in time to influence the transfer decision and there were no delays in the treatment schedule as a result of this study.

### Evaluating the karyotype of the embryo and polar bodies: array-based aneuploidy screening

Polar body and blastomere or trophectoderm (TE) biopsies were processed using SNP array based aneuploidy screening, as previously described [1]. In short, cells were lysed in alkaline solution and underwent whole genome amplification using GenomePlex WGA4 (Sigma Aldrich), followed by SNP array-based analysis of copy number and genotypes using NspI SNP genotyping arrays, copy number analysis tool, and GTYPE software (Affymetrix) [2]. Resulting karyotype predictions of the embryos and corresponding polar body pairs were compared to determine the ability of polar body based aneuploidy screening to predict embryo ploidy.

### DNA fingerprinting to determine clinical outcomes

Parental genomic DNA was genotyped on the NspI array as recommended by the supplier (Affymetrix) and used to identify informative SNPs for the conceptus, embryonic, and polar body derived DNA, as previously described [17, 18]. Once the genotype of the conceptus was known, the results were compared with the genotype of the transferred embryos and the corresponding polar bodies to determine which embryos implanted and progressed through delivery [17, 18].

### Evaluating the predictive value of polar body-based aneuploidy screening for embryo ploidy

Straightforward, descriptive statistics were applied to calculate the predictive value of both polar bodies for embryo ploidy. First, we predicted the chromosomal status of each embryo based on the ploidy of its corresponding polar bodies. When both polar bodies were euploid, the embryo was predicted to be euploid. When at least one polar body was aneuploid, the embryo was predicted to be aneuploid. If one of the two polar bodies failed to amplify or had no diagnosis and the other was aneuploid, the embryo was defined as aneuploid, despite not having a result in the other polar body. Conversely, an embryo with one euploid polar body, but no result in the other, was defined as unknown and excluded from the predictive analysis. Embryos whose DNA failed to amplify or in which a karyotype prediction could not be accurately made were excluded from the predictive analysis, along with their corresponding polar bodies. The positive predictive value was calculated by dividing the sum of euploid polar body outcomes corresponding to euploid embryos (true positives) by the total number of euploid polar body outcomes (true positives + false positives). The negative predictive value was calculated by dividing the sum of aneuploid polar body outcomes corresponding to aneuploid embryos (true negatives) by the total number of aneuploid polar body outcomes (true negatives + false negatives). The overall embryo ploidy predictive value of both polar bodies was calculated by dividing the sum of euploid polar bodies corresponding to euploid embryos and aneuploid polar bodies corresponding to aneuploid embryos by the total number of aneuploid and euploid outcomes. The final results are expressed as a percentage.

### Evaluating the predictive values of polar body-based aneuploidy screening for clinical outcome

To calculate the predictive values of euploid and aneuploid polar body-based aneuploidy screening results for clinical outcome, we used identical methods to those previously detailed for aneuploidy screening in embryo biopsy [2]. Briefly, the outcomes for each embryo were determined. Embryos that implanted and progressed through delivery were said to have a successful outcome or a sustained implantation. All other embryos, whether they failed to implant or resulted in a biochemical or clinical loss, were considered to have failed.

The predictive value of an aneuploid result was calculated by dividing the total number of embryos that had been designated as aneuploid by polar body based aneuploidy screening and that failed to implant by the total number of embryos transferred that had aneuploid polar body screening results (aneuploid polar body failed/all with aneuploid polar bodies). The predictive value of a euploid result was calculated by dividing the number of predicted euploid embryos that had sustained implantation by the total number of embryos that were designated as having normal genetics by polar body based aneuploidy screening (euploid polar body implanted/all with euploid polar bodies). The results are expressed as a percentage.

Additional analyses included evaluating the predictive values of euploid and aneuploid screening results in polar bodies from day 3 (cleavage-stage) embryo transfers as opposed to those from day 5 (blastocyst-stage) transfers. Finally, we compared the clinical predictive value for sustained implantation from euploid and aneuploid polar body based screening results to the predictive values obtained from direct embryo based aneuploidy screening. Chi-squared analyses were performed and an α-error of <0.05 was considered significant for all comparisons.

## Results

### Reliability of obtaining a result

Ninety-six patients with a mean maternal age of 35.3 ± 4.6 years participated in this study. A total of 459 embryos were evaluated. Nine (2 %) of the first polar body samples failed to amplify and 10 (2 %) amplified but were nonconcurrent as previously defined [1]. Of the 440 (96 %) evaluable first polar body array results, 330 (75 %) were euploid and 110 (25 %) were aneuploid. Thirty two (7 %) of second polar body samples failed to amplify and 5 (1 %) amplified but were nonconcurrent. Of the 422 (92 %) evaluable second polar body array results, 320 (76 %) were euploid and 102 (24 %) were aneuploid. Seventeen (4 %) of the embryo samples failed to amplify and 9 (2 %) were nonconcurrent. Of the 433 (94 %) evaluable array results, 254 (59 %) were euploid and 179 (41 %) were aneuploid. Of the samples with discrepant polar body and embryo biopsy diagnoses, there was not a general pattern to characterize the discrepancies; i.e. some cases predicted an isolated monosomy, isolated trisomy, or complex aneuploidy in an embryo with a euploid biopsy result. Failure to obtain a result using a polar body approach was significantly higher than direct testing of the embryo (12.2 % vs. 5.7 %; RR 2.2, 95 % CI 1.4–3.4, *P* < 0.001).

### Predictive value for embryo ploidy

We next analyzed the level of agreement between polar body and embryo based ploidy results. When both polar bodies were euploid, the resulting embryo was euploid in 74.7 % (174/233) of cases. When the array results from the polar bodies predicted aneuploidy, 63.9 % (101/158) of embryos were aneuploid. Incorporating both the negative and positive predictive values, the overall predictive value of polar body-based aneuploidy screening was determined to be 70.3 %.

### Predictive value for implantation

**Fig. 1:**
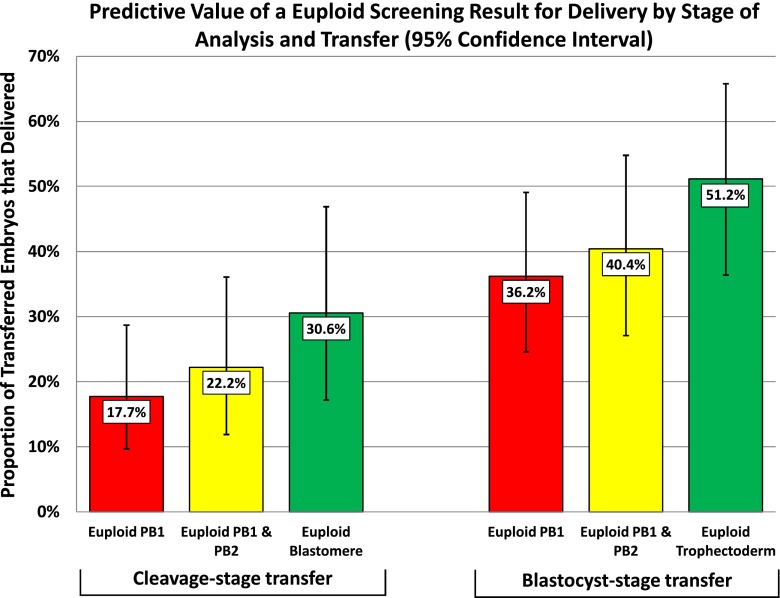
The sustained implantation predictive value (with 95 % confidence interval) of a euploid screening result obtained from the first polar body (PB1), PB1 and the second polar body (PB2), or a direct embryo biopsy for each stage of embryo transfer (cleavage-stage and blastocyst stage)

Embryos with euploid first polar body and second polar body had sustained implantation rates that were not significantly higher than the overall cohort transferred without aneuploidy screening (32 % vs. 23 %, *P* = 0.16). In contrast, embryos with a euploid embryo biopsy had sustained implantation rates that were significantly higher than the overall cohort transferred without aneuploidy screening (42 % vs. 23 %, *P* = 0.003). The predictive value of a euploid embryo biopsy was higher than a euploid polar body biopsy at each stage of transfer, with direct analysis of trophectoderm having the highest predictive value for delivery (Fig. 1).

## Discussion

In theory, polar body CCS offers potential to improve patient outcomes by reducing the time to pregnancy and the incidence of miscarriage. However, the efficacy is contingent on the ability to successfully predict the chromosomal status of the embryo. The present work reveals that polar body aneuploidy screening, using a method with 98.6 % diagnostic accuracy [1], is limited in its ability to predict subsequent embryo ploidy based upon consistency with blastomere and trophectoderm analysis (70.3 %). Some prior studies which assessed performance of polar body based screening only compared results with ensuing whole embryos where aneuploidy from post-zygotic mitotic errors would not be detected [13, 19]. That is, evaluating the whole embryo would mask all mitotic nondisjunction errors since the total number of chromosomes within the entire embryo would be balanced. This analysis strategy would therefore bias the results by only detecting aneuploidy of meiotic origin, and favor finding a correlation between the polar body and the embryo, since polar bodies only allow detection of maternal meiotic errors. In contrast, the present study evaluated cleavage-stage and trophectoderm biopsies thus providing an opportunity to detect aneuploidy derived from mitotic errors and providing a more comprehensive assessment of all origins of embryonic aneuploidy.

Several challenges associated with polar body testing itself may result in the inability to predict the ploidy of the subsequent embryo. First, according to current practice, an embryo is diagnosed as abnormal based on the observation of aneuploidy in either polar body or in first polar body alone, irrespective of whether the observed error is reciprocal [20, 21]. In cases of reciprocal aneuploidy, when a meiosis I error due to premature separation of sister chromatids (PSSC) compensates in meiosis II, the embryo should be disomic for the reciprocal aneuploid chromosome. Isolated reciprocal aneuploidies due to PSSC represent a potential source of misdiagnosis with polar body testing. Given that (i) reciprocal aneuploidy is known to occur frequently in polar bodies observed during human IVF [9, 22], (ii) PSSC is the predominant mechanism of MI error [23, 24], and (iii) the delivery of a healthy child has been documented from an oocyte with reciprocal aneuploid polar bodies [25], the use of polar bodies for aneuploidy screening raises immediate practical concerns, as it may exclude embryos with true reproductive potential.

While the embryo ploidy predictive value of a euploid polar body diagnosis (74.7 %) is superior to that of an aneuploid diagnosis (63.9 %), it still implies that 1 in 4 embryos diagnosed as normal by polar body-based aneuploidy screening will be aneuploid. This may limit its ability to improve IVF outcomes. One source of this error is that polar body testing can only evaluate the maternal contribution to aneuploidy. Although the majority of embryonic aneuploidies are maternal in origin, paternal and post-fertilization contributions are estimated to account for about 10 % of embryonic abnormalities in clinical miscarriages [10] and possibly a higher proportion of preimplantation embryos that fail to implant [26–28].

The findings of the present work differ from data published by Geraedts et al. in the preclinical study of polar body aCGH for prediction of zygote ploidy [13]. The ESHRE aneuploidy screening Task Force aCGH data indicated an aneuploid polar body result was 94 % predictive of the ploidy status of the zygote [13]. However, the aCGH methodology used had not been evaluated for accuracy in predicting aneuploidy from single cells with previously characterized karyotypes. In contrast, the present study involved an independent CCS platform utilizing a validated, WGA and SNP array technology [1, 3, 29]. SNP array data reveals an aneuploid polar body diagnosis is only 63.9 % predictive of embryo ploidy. Several factors may account for this discrepancy, including the fact that the ESHRE study tested early zygotes before the cleavage stage of development when mitotic errors might occur.

Other reasons for divergent findings from the ESHRE study may relate to study size. The present analysis includes 381 paired polar body and embryo samples, which exceeds the sample size of the ESHRE analysis (138 oocyte/polar body pairs) [13]. Differences between each study’s patient populations also must be considered. The mean maternal age in the current investigation is 35.3 +/− 4.6, while the ESHRE study evaluated patients with an average age of 40 [13]. Most significantly, Geraedts et al. reported an overall embryonic aneuploidy rate of 76 %, whereas we report an aneuploidy rate of only 41 %.

The present study indicates that polar body aneuploidy screening has limited ability to predict embryo ploidy. However, it remains to be seen if this level of accuracy is sufficient to improve IVF clinical outcomes. The sample size of embryos transferred with known reproductive outcome did not demonstrate a significant improvement when using polar body-based screening compared with transferring the entire cohort had it been unscreened. A euploid result on direct embryo biopsy, on the contrary, would result in a significant improvement in sustained implantation rates. The positive predictive values in Fig. 1 suggest that trophectoderm biopsy of the blastocyst may be the optimal stage in embryonic development for aneuploidy screening.

A normal result with trophectoderm biopsy is known to be more predictive of a positive clinical outcome than blastomere biopsy [2]. Even more, it has been shown that blastomere biopsy significantly impairs embryonic reproductive potential while trophectoderm biopsy does not [30]. The current study evaluates polar body testing as a proposed alternative, but concludes that polar body testing is limited by imprecision. Lending strong support to this conclusion, a recent study assessing the optimal biopsy stage for aneuploidy screening found testing at the polar body stage to be least accurate, due to the high-incidence of post-zygotic events [15]. Furthermore, another study using aCGH found a 12 % false positive rate when comparing predicted aneuploid chromosomes in the polar bodies to whole embryos, despite the lack of ability to detect differences from mitotic derived aneuploidies [16]. In addition to reduced predictive value, our data also indicate reduced reliability of obtaining a diagnosis, with a 12.2 % no result rate when testing 2 polar bodies per embryo, as compared to a 5.7 % no result rate with embryo biopsy alone. Therefore, the final realization of benefit from aneuploidy screening may require trophectoderm biopsy at the blastocyst-stage.
